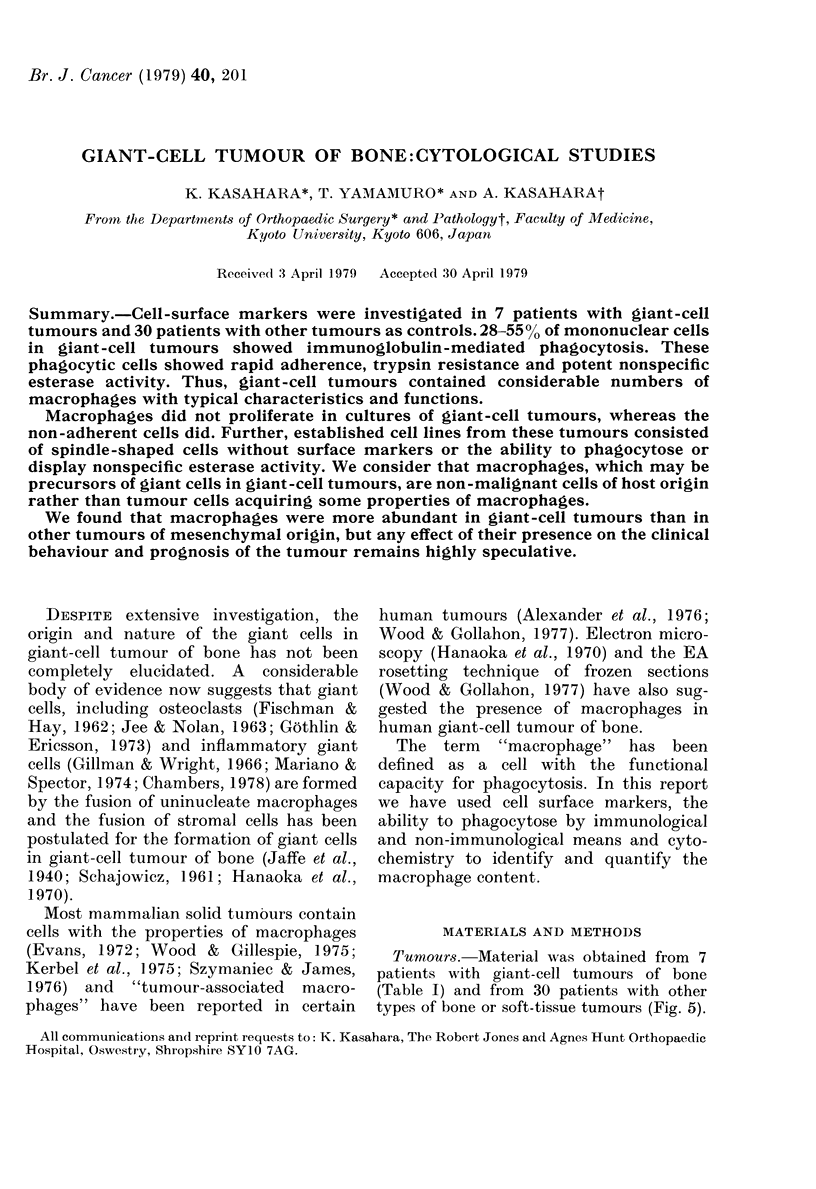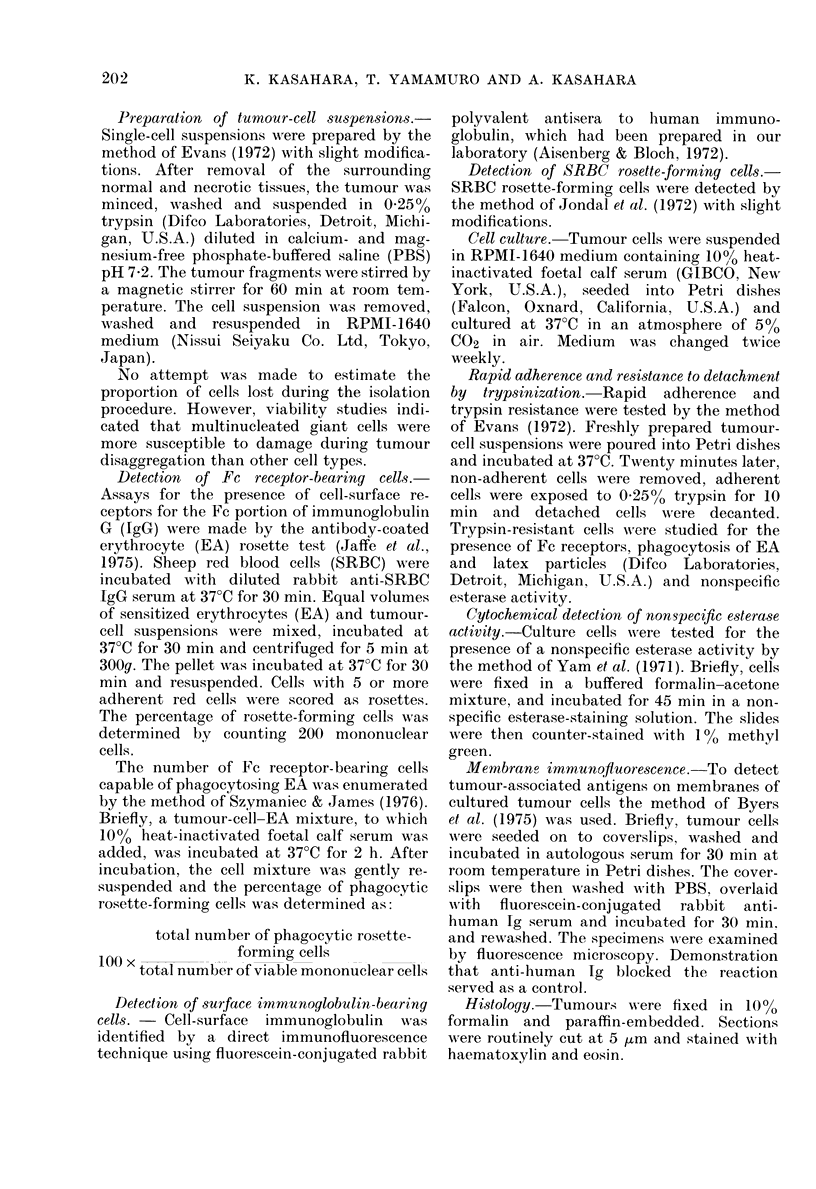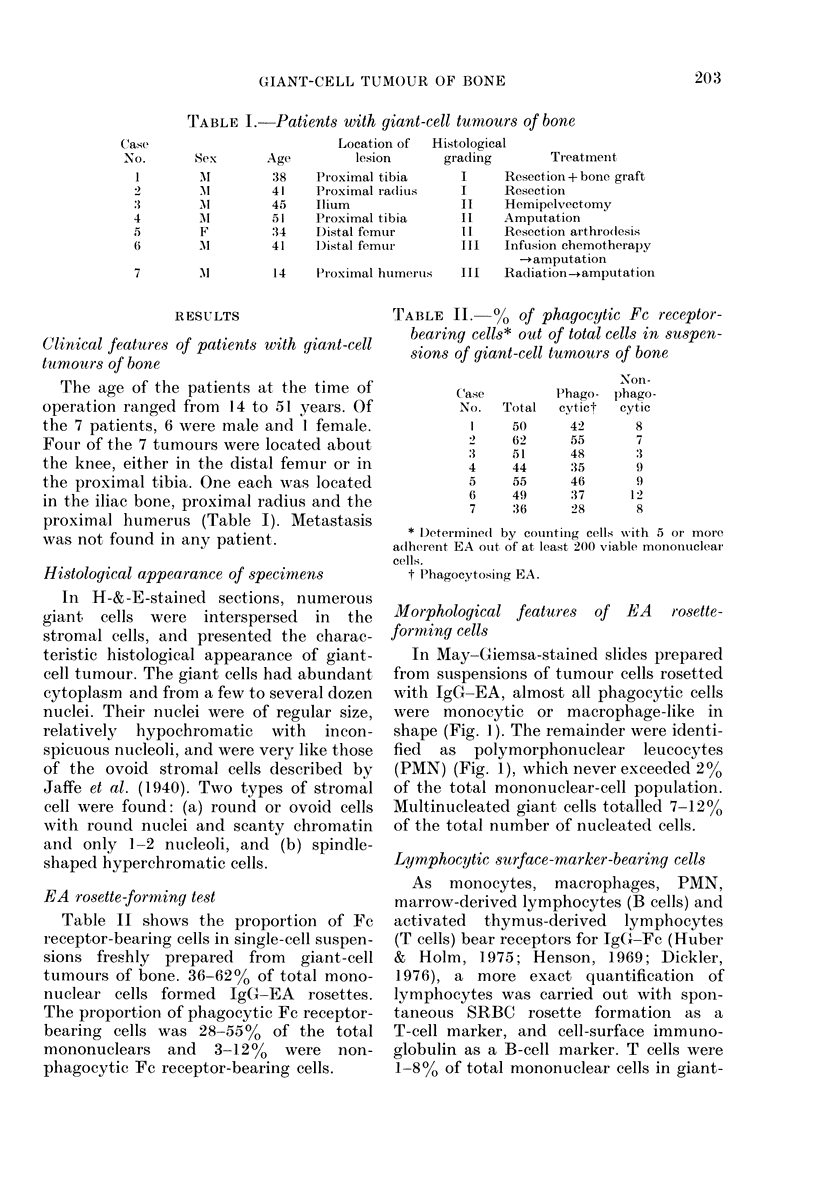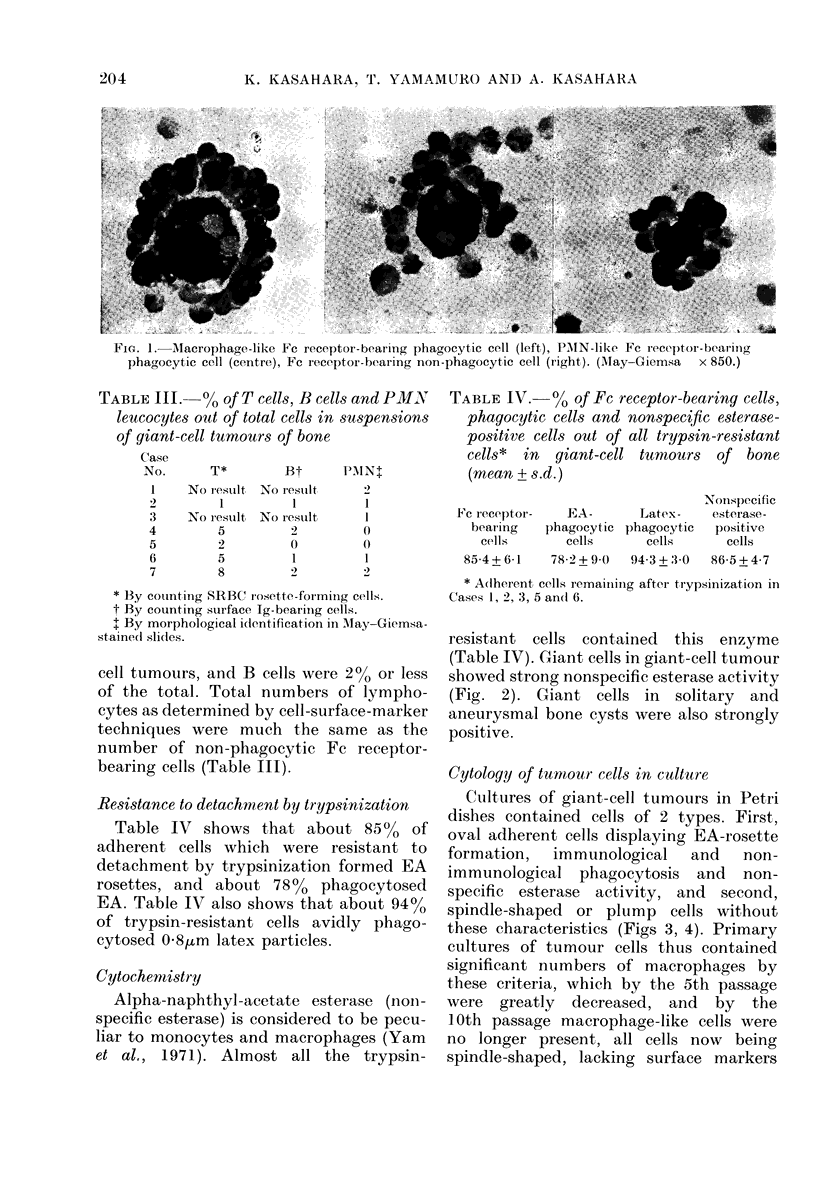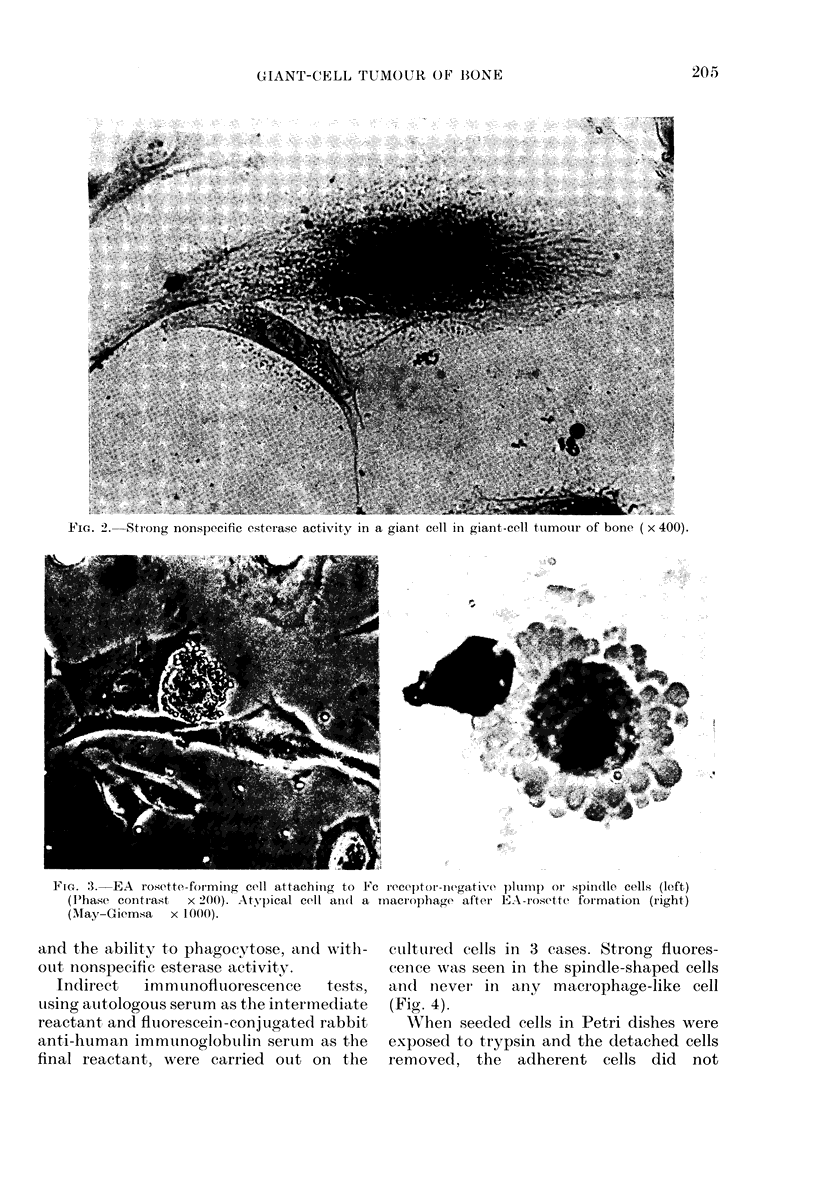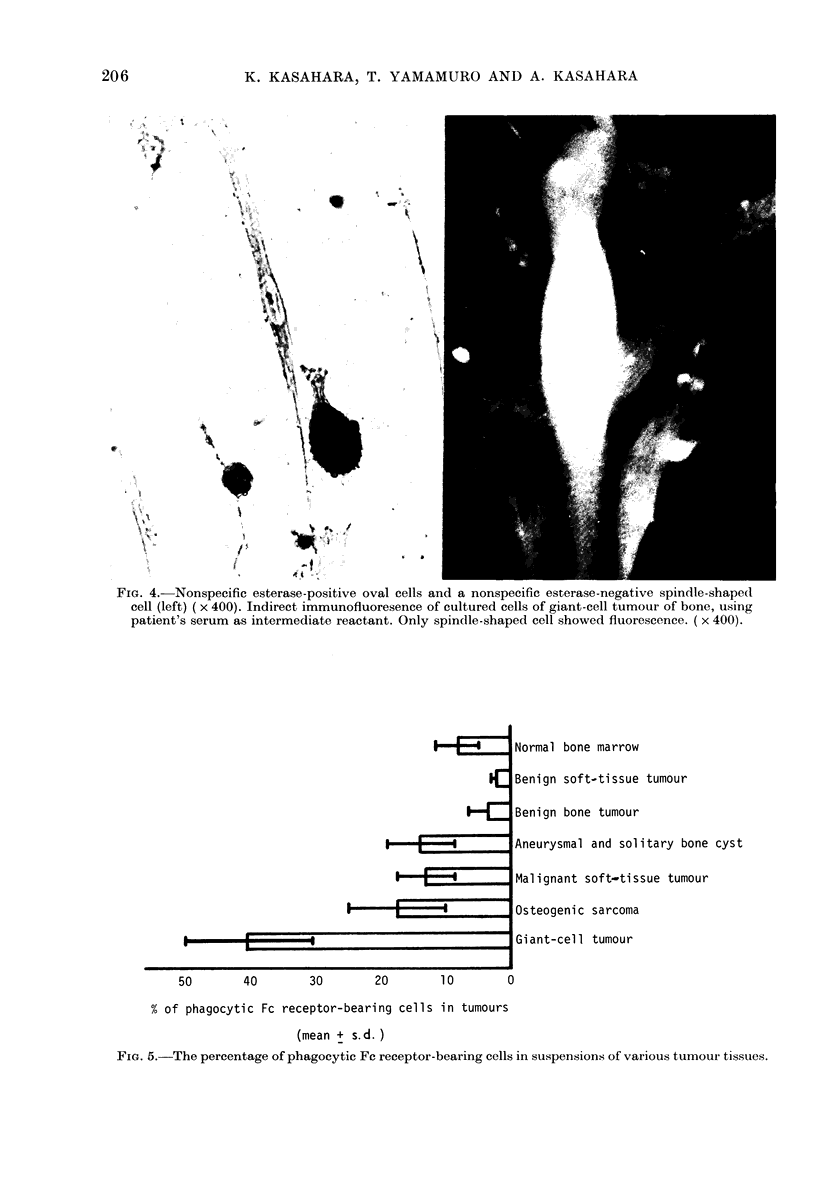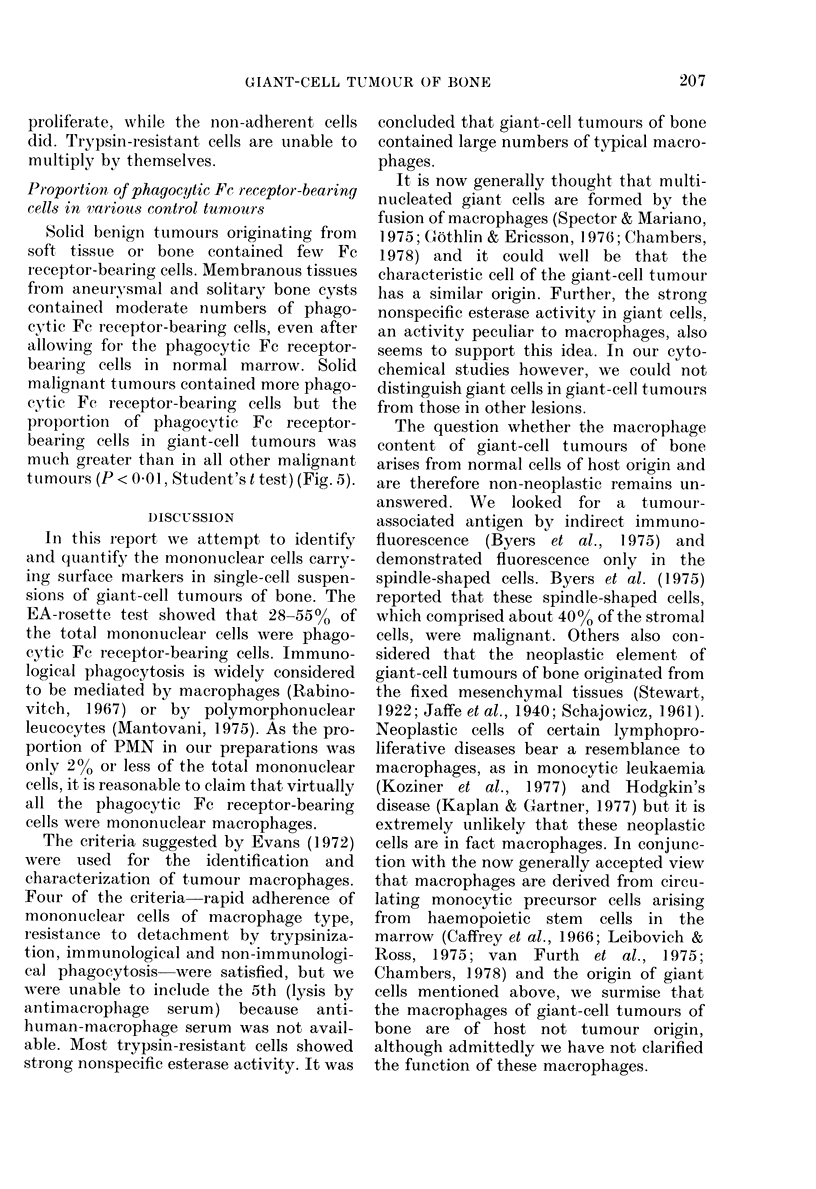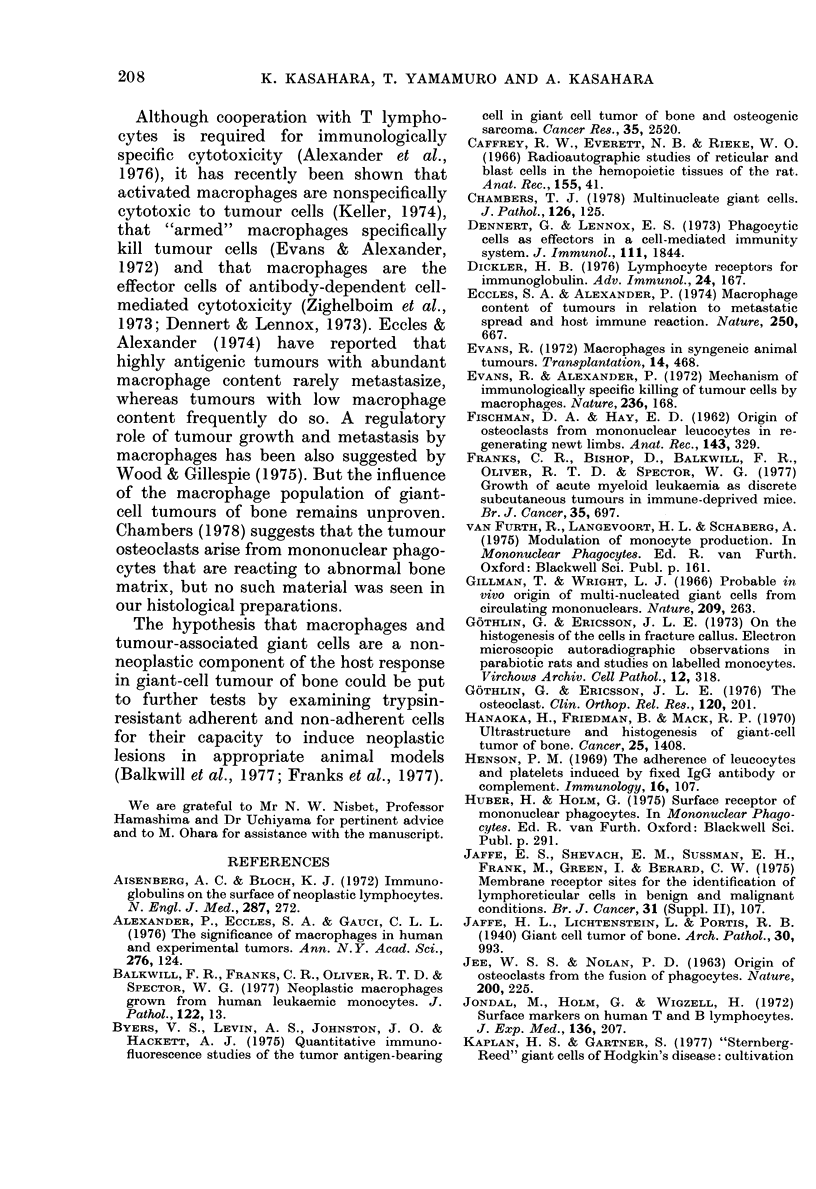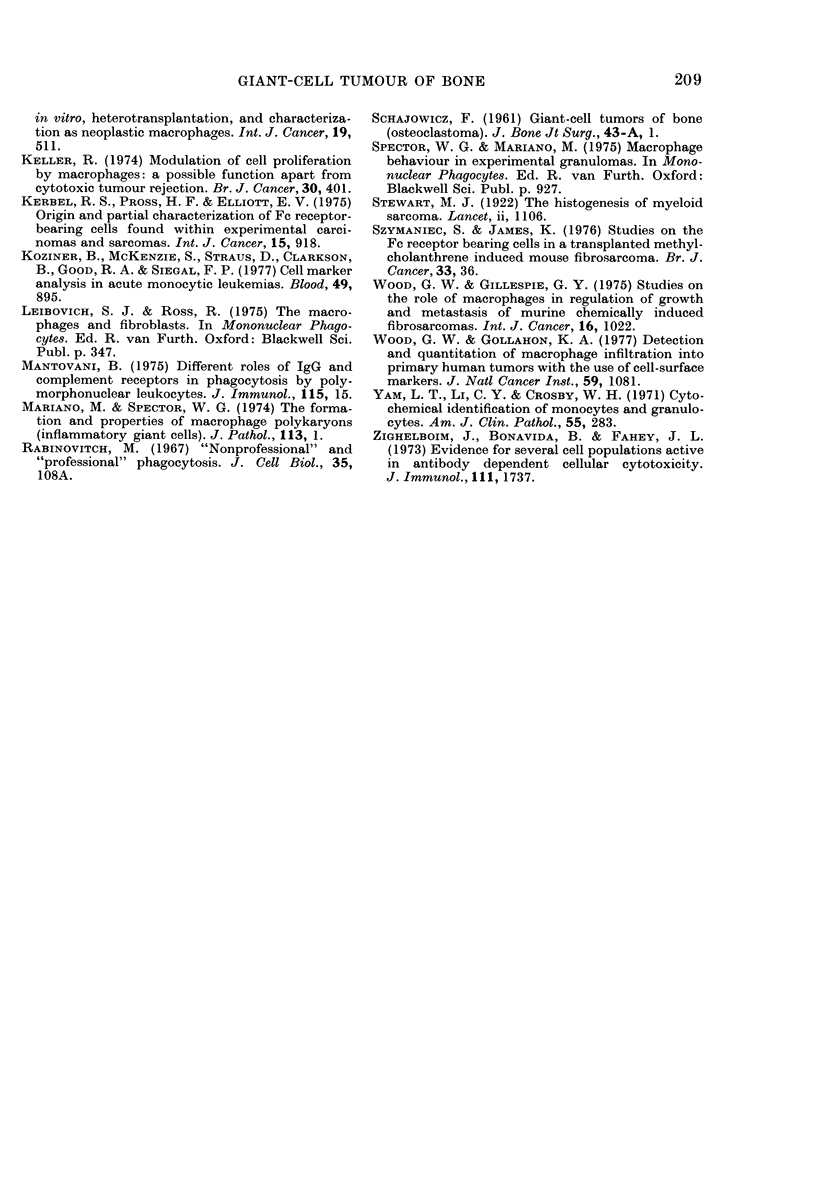# Giant-cell tumour of bone: cytological studies.

**DOI:** 10.1038/bjc.1979.167

**Published:** 1979-08

**Authors:** K. Kasahara, T. Yamamuro, A. Kasahara

## Abstract

**Images:**


*Br. J. Cancer* (1979) **40**, 201

### GIANT-CELL TUMOUR OF BONE: CYTOLOGICAL STUDIES

K. KASAHARA*, T. YAMAMURO* AND A. KASAHARA†

*From the Departments of Orthopaedic Surgery* and Pathology†, Faculty of Medicine, Kyoto University, Kyoto* 606, *Japan*

Received 3 April 1979 Accepted 30 April 1979

**Summary.—Cell-surface markers were investigated in 7 patients with giant-cell tumours and 30 patients with other tumours as controls. 28–55% of mononuclear cells in giant-cell tumours showed immunoglobulin-mediated phagocytosis. These phagocytic cells showed rapid adherence, trypsin resistance and potent nonspecific esterase activity. Thus, giant-cell tumours contained considerable numbers of macrophages with typical characteristics and functions.**

**Macrophages did not proliferate in cultures of giant-cell tumours, whereas the non-adherent cells did. Further, established cell lines from these tumours consisted of spindle-shaped cells without surface markers or the ability to phagocytose or display nonspecific esterase activity. We consider that macrophages, which may be precursors of giant cells in giant-cell tumours, are non-malignant cells of host origin rather than tumour cells acquiring some properties of macrophages.**

**We found that macrophages were more abundant in giant-cell tumours than in other tumours of mesenchymal origin, but any effect of their presence on the clinical behaviour and prognosis of the tumour remains highly speculative.**

DESPITE extensive investigation, the origin and nature of the giant cells in giant-cell tumour of bone has not been completely elucidated. A considerable body of evidence now suggests that giant cells, including osteoclasts (Fischman & Hay, 1962; Jee & Nolan, 1963; Göthlin & Ericsson, 1973) and inflammatory giant cells (Gillman & Wright, 1966; Mariano & Spector, 1974; Chambers, 1978) are formed by the fusion of uninucleate macrophages and the fusion of stromal cells has been postulated for the formation of giant cells in giant-cell tumour of bone (Jaffe *et al.*, 1940; Schajowicz, 1961; Hanaoka *et al.*, 1970).

Most mammalian solid tumours contain cells with the properties of macrophages (Evans, 1972; Wood & Gillespie, 1975; Kerbel *et al.*, 1975; Szymaniec & James, 1976) and "tumour-associated macrophages" have been reported in certain human tumours (Alexander *et al.*, 1976; Wood & Gollahon, 1977). Electron microscopy (Hanaoka *et al.*, 1970) and the EA rosetting technique of frozen sections (Wood & Gollahon, 1977) have also suggested the presence of macrophages in human giant-cell tumour of bone.

The term "macrophage" has been defined as a cell with the functional capacity for phagocytosis. In this report we have used cell surface markers, the ability to phagocytose by immunological and non-immunological means and cytochemistry to identify and quantify the macrophage content.

### MATERIALS AND METHODS

*Tumours.*—Material was obtained from 7 patients with giant-cell tumours of bone (Table I) and from 30 patients with other types of bone or soft-tissue tumours (Fig. 5).

All communications and reprint requests to: K. Kasahara, The Robert Jones and Agnes Hunt Orthopaedic Hospital, Oswestry, Shropshire SY10 7AG.

*Preparation of tumour-cell suspensions.*—Single-cell suspensions were prepared by the method of Evans (1972) with slight modifications. After removal of the surrounding normal and necrotic tissues, the tumour was minced, washed and suspended in 0·25% trypsin (Difco Laboratories, Detroit, Michigan, U.S.A.) diluted in calcium- and magnesium-free phosphate-buffered saline (PBS) pH 7·2. The tumour fragments were stirred by a magnetic stirrer for 60 min at room temperature. The cell suspension was removed, washed and resuspended in RPMI-1640 medium (Nissui Seiyaku Co. Ltd, Tokyo, Japan).

No attempt was made to estimate the proportion of cells lost during the isolation procedure. However, viability studies indicated that multinucleated giant cells were more susceptible to damage during tumour disaggregation than other cell types.

*Detection of Fc receptor-bearing cells.*—Assays for the presence of cell-surface receptors for the Fc portion of immunoglobulin G (IgG) were made by the antibody-coated erythrocyte (EA) rosette test (Jaffe *et al.*, 1975). Sheep red blood cells (SRBC) were incubated with diluted rabbit anti-SRBC IgG serum at 37°C for 30 min. Equal volumes of sensitized erythrocytes (EA) and tumour-cell suspensions were mixed, incubated at 37°C for 30 min and centrifuged for 5 min at 300*g*. The pellet was incubated at 37°C for 30 min and resuspended. Cells with 5 or more adherent red cells were scored as rosettes. The percentage of rosette-forming cells was determined by counting 200 mononuclear cells.

The number of Fc receptor-bearing cells capable of phagocytosing EA was enumerated by the method of Szymaniec & James (1976). Briefly, a tumour-cell–EA mixture, to which 10% heat-inactivated foetal calf serum was added, was incubated at 37°C for 2 h. After incubation, the cell mixture was gently resuspended and the percentage of phagocytic rosette-forming cells was determined as:

$$100 \times \frac{\text{total number of phagocytic rosette-forming cells}}{\text{total number of viable mononuclear cells}}$$

*Detection of surface immunoglobulin-bearing cells.* — Cell-surface immunoglobulin was identified by a direct immunofluorescence technique using fluorescein-conjugated rabbit polyvalent antisera to human immunoglobulin, which had been prepared in our laboratory (Aisenberg & Bloch, 1972).

*Detection of SRBC rosette-forming cells.*—SRBC rosette-forming cells were detected by the method of Jondal *et al.* (1972) with slight modifications.

*Cell culture.*—Tumour cells were suspended in RPMI-1640 medium containing 10% heat-inactivated foetal calf serum (GIBCO, New York, U.S.A.), seeded into Petri dishes (Falcon, Oxnard, California, U.S.A.) and cultured at 37°C in an atmosphere of 5% $CO_2$ in air. Medium was changed twice weekly.

*Rapid adherence and resistance to detachment by trypsinization.*—Rapid adherence and trypsin resistance were tested by the method of Evans (1972). Freshly prepared tumour-cell suspensions were poured into Petri dishes and incubated at 37°C. Twenty minutes later, non-adherent cells were removed, adherent cells were exposed to 0·25% trypsin for 10 min and detached cells were decanted. Trypsin-resistant cells were studied for the presence of Fc receptors, phagocytosis of EA and latex particles (Difco Laboratories, Detroit, Michigan, U.S.A.) and nonspecific esterase activity.

*Cytochemical detection of nonspecific esterase activity.*—Culture cells were tested for the presence of a nonspecific esterase activity by the method of Yam *et al.* (1971). Briefly, cells were fixed in a buffered formalin–acetone mixture, and incubated for 45 min in a nonspecific esterase-staining solution. The slides were then counter-stained with 1% methyl green.

*Membrane immunofluorescence.*—To detect tumour-associated antigens on membranes of cultured tumour cells the method of Byers *et al.* (1975) was used. Briefly, tumour cells were seeded on to coverslips, washed and incubated in autologous serum for 30 min at room temperature in Petri dishes. The coverslips were then washed with PBS, overlaid with fluorescein-conjugated rabbit anti-human Ig serum and incubated for 30 min, and rewashed. The specimens were examined by fluorescence microscopy. Demonstration that anti-human Ig blocked the reaction served as a control.

*Histology.*—Tumours were fixed in 10% formalin and paraffin-embedded. Sections were routinely cut at 5 $\mu$m and stained with haematoxylin and eosin.

TABLE I.—*Patients with giant-cell tumours of bone*

| Case No. | Sex | Age | Location of lesion | Histological grading | Treatment |
|---|---|---|---|---|---|
| 1 | M | 38 | Proximal tibia | I | Resection + bone graft |
| 2 | M | 41 | Proximal radius | I | Resection |
| 3 | M | 45 | Ilium | II | Hemipelvectomy |
| 4 | M | 51 | Proximal tibia | II | Amputation |
| 5 | F | 34 | Distal femur | II | Resection arthrodesis |
| 6 | M | 41 | Distal femur | III | Infusion chemotherapy →amputation |
| 7 | M | 14 | Proximal humerus | III | Radiation→amputation |

### RESULTS

### *Clinical features of patients with giant-cell tumours of bone*

The age of the patients at the time of operation ranged from 14 to 51 years. Of the 7 patients, 6 were male and 1 female. Four of the 7 tumours were located about the knee, either in the distal femur or in the proximal tibia. One each was located in the iliac bone, proximal radius and the proximal humerus (Table I). Metastasis was not found in any patient.

### *Histological appearance of specimens*

In H-&-E-stained sections, numerous giant cells were interspersed in the stromal cells, and presented the characteristic histological appearance of giant-cell tumour. The giant cells had abundant cytoplasm and from a few to several dozen nuclei. Their nuclei were of regular size, relatively hypochromatic with inconspicuous nucleoli, and were very like those of the ovoid stromal cells described by Jaffe *et al.* (1940). Two types of stromal cell were found: (a) round or ovoid cells with round nuclei and scanty chromatin and only 1–2 nucleoli, and (b) spindle-shaped hyperchromatic cells.

### *EA rosette-forming test*

Table II shows the proportion of Fc receptor-bearing cells in single-cell suspensions freshly prepared from giant-cell tumours of bone. 36–62% of total mononuclear cells formed IgG–EA rosettes. The proportion of phagocytic Fc receptor-bearing cells was 28–55% of the total mononuclears and 3–12% were non-phagocytic Fc receptor-bearing cells.

TABLE II.—*% of phagocytic Fc receptor-bearing cells* out of total cells in suspensions of giant-cell tumours of bone*

| Case No. | Total | Phagocytic† | Non-phagocytic |
|---|---|---|---|
| 1 | 50 | 42 | 8 |
| 2 | 62 | 55 | 7 |
| 3 | 51 | 48 | 3 |
| 4 | 44 | 35 | 9 |
| 5 | 55 | 46 | 9 |
| 6 | 49 | 37 | 12 |
| 7 | 36 | 28 | 8 |

* Determined by counting cells with 5 or more adherent EA out of at least 200 viable mononuclear cells.
† Phagocytosing EA.

### *Morphological features of EA rosette-forming cells*

In May–Giemsa-stained slides prepared from suspensions of tumour cells rosetted with IgG–EA, almost all phagocytic cells were monocytic or macrophage-like in shape (Fig. 1). The remainder were identified as polymorphonuclear leucocytes (PMN) (Fig. 1), which never exceeded 2% of the total mononuclear-cell population. Multinucleated giant cells totalled 7–12% of the total number of nucleated cells.

### *Lymphocytic surface-marker-bearing cells*

As monocytes, macrophages, PMN, marrow-derived lymphocytes (B cells) and activated thymus-derived lymphocytes (T cells) bear receptors for IgG–Fc (Huber & Holm, 1975; Henson, 1969; Dickler, 1976), a more exact quantification of lymphocytes was carried out with spontaneous SRBC rosette formation as a T-cell marker, and cell-surface immunoglobulin as a B-cell marker. T cells were 1–8% of total mononuclear cells in giant-

FIG. 1.—Macrophage-like Fc receptor-bearing phagocytic cell (left), PMN-like Fc receptor-bearing phagocytic cell (centre), Fc receptor-bearing non-phagocytic cell (right). (May–Giemsa ×850.)

TABLE III.—% *of T cells, B cells and PMN leucocytes out of total cells in suspensions of giant-cell tumours of bone*

| Case No. | T* | B† | PMN‡ |
|---|---|---|---|
| 1 | No result | No result | 2 |
| 2 | 1 | 1 | 1 |
| 3 | No result | No result | 1 |
| 4 | 5 | 2 | 0 |
| 5 | 2 | 0 | 0 |
| 6 | 5 | 1 | 1 |
| 7 | 8 | 2 | 2 |

* By counting SRBC rosette-forming cells.
† By counting surface Ig-bearing cells.
‡ By morphological identification in May–Giemsa-stained slides.

cell tumours, and B cells were 2% or less of the total. Total numbers of lymphocytes as determined by cell-surface-marker techniques were much the same as the number of non-phagocytic Fc receptor-bearing cells (Table III).

*Resistance to detachment by trypsinization*

Table IV shows that about 85% of adherent cells which were resistant to detachment by trypsinization formed EA rosettes, and about 78% phagocytosed EA. Table IV also shows that about 94% of trypsin-resistant cells avidly phagocytosed 0·8$\mu$m latex particles.

*Cytochemistry*

Alpha-naphthyl-acetate esterase (nonspecific esterase) is considered to be peculiar to monocytes and macrophages (Yam *et al.*, 1971). Almost all the trypsin-resistant cells contained this enzyme (Table IV). Giant cells in giant-cell tumour showed strong nonspecific esterase activity (Fig. 2). Giant cells in solitary and aneurysmal bone cysts were also strongly positive.

TABLE IV.—% *of Fc receptor-bearing cells, phagocytic cells and nonspecific esterase-positive cells out of all trypsin-resistant cells* in giant-cell tumours of bone (mean* ± *s.d.)*

| Fc receptor-bearing cells | EA-phagocytic cells | Latex-phagocytic cells | Nonspecific esterase-positive cells |
|---|---|---|---|
| 85·4 ± 6·1 | 78·2 ± 9·0 | 94·3 ± 3·0 | 86·5 ± 4·7 |

* Adherent cells remaining after trypsinization in Cases 1, 2, 3, 5 and 6.

*Cytology of tumour cells in culture*

Cultures of giant-cell tumours in Petri dishes contained cells of 2 types. First, oval adherent cells displaying EA-rosette formation, immunological and non-immunological phagocytosis and non-specific esterase activity, and second, spindle-shaped or plump cells without these characteristics (Figs 3, 4). Primary cultures of tumour cells thus contained significant numbers of macrophages by these criteria, which by the 5th passage were greatly decreased, and by the 10th passage macrophage-like cells were no longer present, all cells now being spindle-shaped, lacking surface markers

Fig. 2.—Strong nonspecific esterase activity in a giant cell in giant-cell tumour of bone ( × 400).

Fig. 3.—EA rosette-forming cell attaching to Fc receptor-negative plump or spindle cells (left) (Phase contrast × 200). Atypical cell and a macrophage after EA-rosette formation (right) (May–Giemsa × 1000).

and the ability to phagocytose, and without nonspecific esterase activity.

Indirect immunofluorescence tests, using autologous serum as the intermediate reactant and fluorescein-conjugated rabbit anti-human immunoglobulin serum as the final reactant, were carried out on the cultured cells in 3 cases. Strong fluorescence was seen in the spindle-shaped cells and never in any macrophage-like cell (Fig. 4).

When seeded cells in Petri dishes were exposed to trypsin and the detached cells removed, the adherent cells did not

FIG. 4.—Nonspecific esterase-positive oval cells and a nonspecific esterase-negative spindle-shaped cell (left) ( × 400). Indirect immunofluoresence of cultured cells of giant-cell tumour of bone, using patient's serum as intermediate reactant. Only spindle-shaped cell showed fluorescence. ( × 400).

FIG. 5.—The percentage of phagocytic Fc receptor-bearing cells in suspensions of various tumour tissues.

proliferate, while the non-adherent cells did. Trypsin-resistant cells are unable to multiply by themselves.

*Proportion of phagocytic Fc receptor-bearing cells in various control tumours*

Solid benign tumours originating from soft tissue or bone contained few Fc receptor-bearing cells. Membranous tissues from aneurysmal and solitary bone cysts contained moderate numbers of phagocytic Fc receptor-bearing cells, even after allowing for the phagocytic Fc receptor-bearing cells in normal marrow. Solid malignant tumours contained more phagocytic Fc receptor-bearing cells but the proportion of phagocytic Fc receptor-bearing cells in giant-cell tumours was much greater than in all other malignant tumours ($P < 0{\cdot}01$, Student's $t$ test) (Fig. 5).

### DISCUSSION

In this report we attempt to identify and quantify the mononuclear cells carrying surface markers in single-cell suspensions of giant-cell tumours of bone. The EA-rosette test showed that 28–55% of the total mononuclear cells were phagocytic Fc receptor-bearing cells. Immunological phagocytosis is widely considered to be mediated by macrophages (Rabinovitch, 1967) or by polymorphonuclear leucocytes (Mantovani, 1975). As the proportion of PMN in our preparations was only 2% or less of the total mononuclear cells, it is reasonable to claim that virtually all the phagocytic Fc receptor-bearing cells were mononuclear macrophages.

The criteria suggested by Evans (1972) were used for the identification and characterization of tumour macrophages. Four of the criteria—rapid adherence of mononuclear cells of macrophage type, resistance to detachment by trypsinization, immunological and non-immunological phagocytosis—were satisfied, but we were unable to include the 5th (lysis by antimacrophage serum) because anti-human-macrophage serum was not available. Most trypsin-resistant cells showed strong nonspecific esterase activity. It was concluded that giant-cell tumours of bone contained large numbers of typical macrophages.

It is now generally thought that multinucleated giant cells are formed by the fusion of macrophages (Spector & Mariano, 1975; Göthlin & Ericsson, 1976; Chambers, 1978) and it could well be that the characteristic cell of the giant-cell tumour has a similar origin. Further, the strong nonspecific esterase activity in giant cells, an activity peculiar to macrophages, also seems to support this idea. In our cytochemical studies however, we could not distinguish giant cells in giant-cell tumours from those in other lesions.

The question whether the macrophage content of giant-cell tumours of bone arises from normal cells of host origin and are therefore non-neoplastic remains unanswered. We looked for a tumour-associated antigen by indirect immunofluorescence (Byers *et al.*, 1975) and demonstrated fluorescence only in the spindle-shaped cells. Byers *et al.* (1975) reported that these spindle-shaped cells, which comprised about 40% of the stromal cells, were malignant. Others also considered that the neoplastic element of giant-cell tumours of bone originated from the fixed mesenchymal tissues (Stewart, 1922; Jaffe *et al.*, 1940; Schajowicz, 1961). Neoplastic cells of certain lymphoproliferative diseases bear a resemblance to macrophages, as in monocytic leukaemia (Koziner *et al.*, 1977) and Hodgkin's disease (Kaplan & Gartner, 1977) but it is extremely unlikely that these neoplastic cells are in fact macrophages. In conjunction with the now generally accepted view that macrophages are derived from circulating monocytic precursor cells arising from haemopoietic stem cells in the marrow (Caffrey *et al.*, 1966; Leibovich & Ross, 1975; van Furth *et al.*, 1975; Chambers, 1978) and the origin of giant cells mentioned above, we surmise that the macrophages of giant-cell tumours of bone are of host not tumour origin, although admittedly we have not clarified the function of these macrophages.

Although cooperation with T lymphocytes is required for immunologically specific cytotoxicity (Alexander *et al.*, 1976), it has recently been shown that activated macrophages are nonspecifically cytotoxic to tumour cells (Keller, 1974), that "armed" macrophages specifically kill tumour cells (Evans & Alexander, 1972) and that macrophages are the effector cells of antibody-dependent cell-mediated cytotoxicity (Zighelboim *et al.*, 1973; Dennert & Lennox, 1973). Eccles & Alexander (1974) have reported that highly antigenic tumours with abundant macrophage content rarely metastasize, whereas tumours with low macrophage content frequently do so. A regulatory role of tumour growth and metastasis by macrophages has been also suggested by Wood & Gillespie (1975). But the influence of the macrophage population of giant-cell tumours of bone remains unproven. Chambers (1978) suggests that the tumour osteoclasts arise from mononuclear phagocytes that are reacting to abnormal bone matrix, but no such material was seen in our histological preparations.

The hypothesis that macrophages and tumour-associated giant cells are a non-neoplastic component of the host response in giant-cell tumour of bone could be put to further tests by examining trypsin-resistant adherent and non-adherent cells for their capacity to induce neoplastic lesions in appropriate animal models (Balkwill *et al.*, 1977; Franks *et al.*, 1977).

We are grateful to Mr N. W. Nisbet, Professor Hamashima and Dr Uchiyama for pertinent advice and to M. Ohara for assistance with the manuscript.

### REFERENCES

AISENBERG, A. C. & BLOCH, K. J. (1972) Immunoglobulins on the surface of neoplastic lymphocytes. *N. Engl. J. Med.*, **287**, 272.

ALEXANDER, P., ECCLES, S. A. & GAUCI, C. L. L. (1976) The significance of macrophages in human and experimental tumors. *Ann. N.Y. Acad. Sci.*, **276**, 124.

BALKWILL, F. R., FRANKS, C. R., OLIVER, R. T. D. & SPECTOR, W. G. (1977) Neoplastic macrophages grown from human leukaemic monocytes. *J. Pathol.*, **122**, 13.

BYERS, V. S., LEVIN, A. S., JOHNSTON, J. O. & HACKETT, A. J. (1975) Quantitative immunofluorescence studies of the tumor antigen-bearing cell in giant cell tumor of bone and osteogenic sarcoma. *Cancer Res.*, **35**, 2520.

CAFFREY, R. W., EVERETT, N. B. & RIEKE, W. O. (1966) Radioautographic studies of reticular and blast cells in the hemopoietic tissues of the rat. *Anat. Rec.*, **155**, 41.

CHAMBERS, T. J. (1978) Multinucleate giant cells. *J. Pathol.*, **126**, 125.

DENNERT, G. & LENNOX, E. S. (1973) Phagocytic cells as effectors in a cell-mediated immunity system. *J. Immunol.*, **111**, 1844.

DICKLER, H. B. (1976) Lymphocyte receptors for immunoglobulin. *Adv. Immunol.*, **24**, 167.

ECCLES, S. A. & ALEXANDER, P. (1974) Macrophage content of tumours in relation to metastatic spread and host immune reaction. *Nature*, **250**, 667.

EVANS, R. (1972) Macrophages in syngeneic animal tumours. *Transplantation*, **14**, 468.

EVANS, R. & ALEXANDER, P. (1972) Mechanism of immunologically specific killing of tumour cells by macrophages. *Nature*, **236**, 168.

FISCHMAN, D. A. & HAY, E. D. (1962) Origin of osteoclasts from mononuclear leucocytes in regenerating newt limbs. *Anat. Rec.*, **143**, 329.

FRANKS, C. R., BISHOP, D., BALKWILL, F. R., OLIVER, R. T. D. & SPECTOR, W. G. (1977) Growth of acute myeloid leukaemia as discrete subcutaneous tumours in immune-deprived mice. *Br. J. Cancer*, **35**, 697.

VAN FURTH, R., LANGEVOORT, H. L. & SCHABERG, A. (1975) Modulation of monocyte production. In *Mononuclear Phagocytes*. Ed. R. van Furth. Oxford: Blackwell Sci. Publ. p. 161.

GILLMAN, T. & WRIGHT, L. J. (1966) Probable *in vivo* origin of multi-nucleated giant cells from circulating mononuclears. *Nature*, **209**, 263.

GÖTHLIN, G. & ERICSSON, J. L. E. (1973) On the histogenesis of the cells in fracture callus. Electron microscopic autoradiographic observations in parabiotic rats and studies on labelled monocytes. *Virchows Archiv. Cell Pathol.*, **12**, 318.

GÖTHLIN, G. & ERICSSON, J. L. E. (1976) The osteoclast. *Clin. Orthop. Rel. Res.*, **120**, 201.

HANAOKA, H., FRIEDMAN, B. & MACK, R. P. (1970) Ultrastructure and histogenesis of giant-cell tumor of bone. *Cancer*, **25**, 1408.

HENSON, P. M. (1969) The adherence of leucocytes and platelets induced by fixed IgG antibody or complement. *Immunology*, **16**, 107.

HUBER, H. & HOLM, G. (1975) Surface receptor of mononuclear phagocytes. In *Mononuclear Phagocytes*. Ed. R. van Furth. Oxford: Blackwell Sci. Publ. p. 291.

JAFFE, E. S., SHEVACH, E. M., SUSSMAN, E. H., FRANK, M., GREEN, I. & BERARD, C. W. (1975) Membrane receptor sites for the identification of lymphoreticular cells in benign and malignant conditions. *Br. J. Cancer*, **31** (Suppl. II), 107.

JAFFE, H. L., LICHTENSTEIN, L. & PORTIS, R. B. (1940) Giant cell tumor of bone. *Arch. Pathol.*, **30**, 993.

JEE, W. S. S. & NOLAN, P. D. (1963) Origin of osteoclasts from the fusion of phagocytes. *Nature*, **200**, 225.

JONDAL, M., HOLM, G. & WIGZELL, H. (1972) Surface markers on human T and B lymphocytes. *J. Exp. Med.*, **136**, 207.

KAPLAN, H. S. & GARTNER, S. (1977) "Sternberg-Reed" giant cells of Hodgkin's disease: cultivation

*in vitro*, heterotransplantation, and characterization as neoplastic macrophages. *Int. J. Cancer*, **19**, 511.

KELLER, R. (1974) Modulation of cell proliferation by macrophages: a possible function apart from cytotoxic tumour rejection. *Br. J. Cancer*, **30**, 401.

KERBEL, R. S., PROSS, H. F. & ELLIOTT, E. V. (1975) Origin and partial characterization of Fc receptor-bearing cells found within experimental carcinomas and sarcomas. *Int. J. Cancer*, **15**, 918.

KOZINER, B., MCKENZIE, S., STRAUS, D., CLARKSON, B., GOOD, R. A. & SIEGAL, F. P. (1977) Cell marker analysis in acute monocytic leukemias. *Blood*, **49**, 895.

LEIBOVICH, S. J. & ROSS, R. (1975) The macrophages and fibroblasts. In *Mononuclear Phagocytes*. Ed. R. van Furth. Oxford: Blackwell Sci. Publ. p. 347.

MANTOVANI, B. (1975) Different roles of IgG and complement receptors in phagocytosis by polymorphonuclear leukocytes. *J. Immunol.*, **115**, 15.

MARIANO, M. & SPECTOR, W. G. (1974) The formation and properties of macrophage polykaryons (inflammatory giant cells). *J. Pathol.*, **113**, 1.

RABINOVITCH, M. (1967) "Nonprofessional" and "professional" phagocytosis. *J. Cell Biol.*, **35**, 108A.

SCHAJOWICZ, F. (1961) Giant-cell tumors of bone (osteoclastoma). *J. Bone Jt Surg.*, **43-A**, 1.

SPECTOR, W. G. & MARIANO, M. (1975) Macrophage behaviour in experimental granulomas. In *Mononuclear Phagocytes*. Ed. R. van Furth. Oxford: Blackwell Sci. Publ. p. 927.

STEWART, M. J. (1922) The histogenesis of myeloid sarcoma. *Lancet*, ii, 1106.

SZYMANIEC, S. & JAMES, K. (1976) Studies on the Fc receptor bearing cells in a transplanted methylcholanthrene induced mouse fibrosarcoma. *Br. J. Cancer*, **33**, 36.

WOOD, G. W. & GILLESPIE, G. Y. (1975) Studies on the role of macrophages in regulation of growth and metastasis of murine chemically induced fibrosarcomas. *Int. J. Cancer*, **16**, 1022.

WOOD, G. W. & GOLLAHON, K. A. (1977) Detection and quantitation of macrophage infiltration into primary human tumors with the use of cell-surface markers. *J. Natl Cancer Inst.*, **59**, 1081.

YAM, L. T., LI, C. Y. & CROSBY, W. H. (1971) Cytochemical identification of monocytes and granulocytes. *Am. J. Clin. Pathol.*, **55**, 283.

ZIGHELBOIM, J., BONAVIDA, B. & FAHEY, J. L. (1973) Evidence for several cell populations active in antibody dependent cellular cytotoxicity. *J. Immunol.*, **111**, 1737.